# Advancing Open‐Access Education for the Surgical Team Worldwide: The Development and Rollout of the United Nations Global Surgery Learning Hub (SURGhub)

**DOI:** 10.1002/wjs.12661

**Published:** 2025-06-20

**Authors:** Eric O'Flynn, Musliu Adetola Tolani, Emilie Joos, Jean O'Sullivan, Dhananjaya Sharma, Sherry M. Wren, Ainhoa Costas Chavarri, Pauline B. Wake, Andrea S. Parker, Jackie Rowles, Lubna Khan, Sara M. Mohan, Sabina M. Siddiqui, Adolfo Leyva‐Alvizo, Richard O. E. Gardner, Rahel Nardos, Luiz Fernando dos Reis Falcão, Claude Martin, Somprakas Basu, Emilio Velis, Anna M. Darelli‐Anderson, Vincenzo Palatella, Andrew Katz, Rebecca Silvers, Harry Papadopoulos, Advait J. Gandhe, Elizabeth Khvatova, Karina Olivo, Michelle N. Odonkor, Arushi Biswas, Francesca Vitucci, Léa Simon, Ines Perić, Sebastian Hofbauer, Noor Alesawy, Juan Carlos Puyana, Geoffrey Ibbotson

**Affiliations:** ^1^ Institute of Global Surgery Royal College of Surgeons in Ireland Dublin Ireland; ^2^ Department of Surgery Ahmadu Bello University Zaria Nigeria; ^3^ Department of Surgery University of British Columbia Vancouver Canada; ^4^ Tallaght University Hospital Dublin Ireland; ^5^ Department of Surgery NSCB Government Medical College Jabalpur Jabalpur India; ^6^ Department of Surgery Stanford University School of Medicine Stanford California USA; ^7^ Department of Surgery Rwanda Military Referral and Teaching Hospital Kigali Rwanda; ^8^ School of Medicine and Health Sciences University of Papua New Guinea Boroko Papua New Guinea; ^9^ Department of Surgery Tenwek Hospital Bomet Kenya; ^10^ School of Nurse Anesthesia Texas Christian University Fort Worth Texas USA; ^11^ Centre for Global Surgery Baylor College of Medicine Houston Texas USA; ^12^ Cork University Maternity Hospital Cork Ireland; ^13^ Division of Pediatric Surgery University of Arkansas for Medical Sciences Spingdale Arkansas USA; ^14^ Department of Clinical Sciences Tecnologico de Monterrey Monterrey Mexico; ^15^ Division of Paediatric Orthopaedics Hospital for Sick Children Toronto Canada; ^16^ Department of Obstetrics Gynecology and Women's Health University of Minnesota Medical School Minneapolis Minnesota USA; ^17^ Department of Anaesthesia Pain and Intensive Care Federal University of São Paulo Paulista Medical School São Paulo Brazil; ^18^ AO Alliance Chur Switzerland; ^19^ Department of General Surgery All India Institute of Medical Sciences‐Rishikesh Rishikesh India; ^20^ Appropedia Foundation San Salvador El Salvador; ^21^ Department of Surgery University of Utah Salt Lake City Utah USA; ^22^ Independent Teaching and Training Specialist Lausanne Switzerland; ^23^ Bristows LLP London UK; ^24^ Orcro Limited London UK; ^25^ Center for Global Nursing University of California San Francisco California USA; ^26^ Medtronic Watford UK; ^27^ Portsmouth Hospitals University NHS Trust Portsmouth UK; ^28^ Bloomberg School of Public Health Johns Hopkins University Baltimore Maryland USA; ^29^ Operation Smile Montréal Canada; ^30^ Johns Hopkins Center for Global Surgery Johns Hopkins University School of Medicine Baltimore Maryland USA; ^31^ Global Surgery Foundation Geneva Switzerland; ^32^ United Nations Institute for Training and Research Geneva Switzerland

**Keywords:** education, global surgery, training

## Abstract

**Problem:**

Training and professional development programs for surgeons, anesthetists, obstetricians, and perioperative nurses in low‐resource settings are often constrained by lack of access to appropriate training materials. Potential learners looking to access online content are faced with internet connectivity issues, difficulties in finding and accessing resources, resources that are inappropriate for their context and training, and opaque content quality control processes.

**Approach:**

The United Nations Global Surgery Learning Hub (SURGhub) was launched on June 28, 2023 to address this need. SURGhub curates high‐quality surgical, anesthetic, obstetric, and perioperative nursing e‐learning courses and makes them freely available on one integrated online platform, optimized for low‐bandwidth settings. It is a product of the global surgery community, powered by over 200 volunteers and anchored in the United Nations.

**Outcomes:**

In little over 18 months since its launch, SURGhub has enrolled 11,451 registered learners from 190 countries. Fifty‐five percent of users are based in low‐ or lower‐middle income countries. Learners can access 76 interactive e‐learning courses, provided by 20 different institutions. Median course user rating is 4.6/5.

**Discussion:**

SURGhub is addressing the needs of underserved surgical learners through innovative, participatory technological solutions. To address the unmet need, SURGhub must expand its educational offering, including through the addition of new content types, personalized learning, and increased provision of content in languages other than English. The translation of SURGhub educational content into improvements in clinical practice and patient outcomes must be measured.

## Graphical abstract

Training and professional development programmes for surgeons, anaesthetists, obstetricians and perioperative nurses in low‐resource settings are often constrained by lack of access to appropriate training materials. The United Nations Global Surgery Learning Hub (SURGhub) was launched on 28th June 2023 to address this need. SURGhub has enrolled 11,451 registered learners, from 190 countries, who can access 76 interactive e‐learning courses, provided by 20 different institutions.

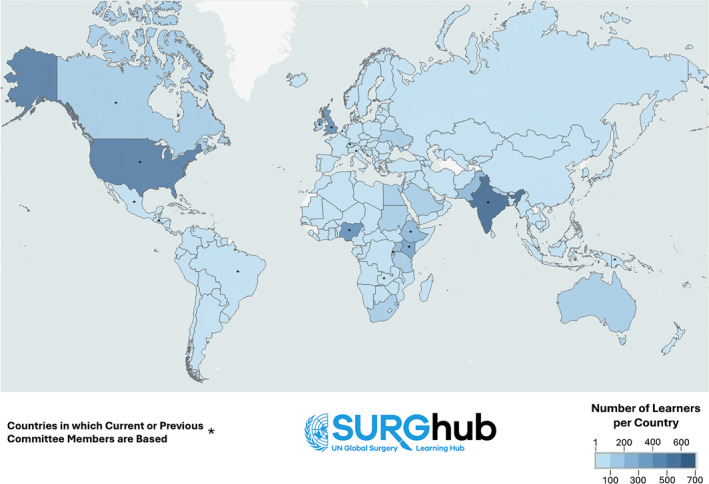

## 1 Problem

Approximately 30% of the global burden of disease can only be effectively addressed by perioperative and surgical care [1], yet the majority of the world's population cannot access safe, affordable surgical and perioperative care when needed [2]. A key challenge is the insufficient number of trained providers: surgeons, anesthetists, obstetricians, and perioperative nurses. Training and professional development programs for these cadres in low‐resource settings are few and are often constrained from scaling up by lack of access to appropriate training materials. Challenges faced by surgical care providers looking to access online educational resources include absent or unstable internet connectivity, a surfeit of resources in some areas and a corresponding dearth in others, and resources that are inappropriate for their context and training. E‐learning content has typically been dispersed across many different platforms, often difficult or impossible for learners to find, presented in inconsistent styles and formats, sometimes costly, and usually without an explicit independent content quality control process [3]. Medical education e‐learning projects in low‐ and middle‐income countries have mainly been “small‐scale” and “short‐termed” [4].

It is difficult to define what constitutes a “low‐resource setting” in healthcare and medical education and training [5]. Although low‐resource settings occur in high‐income countries, and vice versa, there is nevertheless a correlation between gross national income and hospital resources. We can estimate the potential low‐resource setting target audience that would benefit from increased access to surgical education material by quantifying the surgical care workforce in countries defined as low‐ and lower‐middle‐income by the World Bank [6].

In 2015, there were an estimated 441,000 specialist surgeons, anesthetists, and obstetricians in these countries [7]. These specialists likely constitute approximately half of the workforce actually providing surgical care [8]. Furthermore, despite their substantial contribution to the delivery of care, perioperative nurses and those in training for surgical careers were not included in these figures. Therefore, we can extrapolate that the total surgical care workforce in low‐ and lower‐middle‐income countries is likely to exceed one million healthcare workers. Whether in countries with lower national income or elsewhere, there are clearly very many surgical providers with unmet educational needs.

## 2 Approach

With this context in mind, The United Nations Global Surgery Learning Hub (SURGhub, www.surghub.org) was created to address the unmet training, education, and professional development needs of surgical care teams in low‐resource settings. Rather than creating new content, SURGhub seeks to make preexisting content more accessible by curating high‐quality surgical, anesthetic, obstetric, and perioperative nursing e‐learning courses and making them freely available on one integrated online platform.

The SURGhub website was launched on 28^th^ June 2023. It was designed with low‐bandwidth settings in mind, with features including downloadable material, video compression, and video transcripts. Android and iPhone mobile apps were subsequently launched in September 2023, offering greater offline accessibility to content. Users are given the option to register their basic professional and demographic details.

### 2.1 Built by the Global Surgery Community

For SURGhub to meet the learning needs of the global surgery community, it must be a product of the global surgery community. The process of identifying and curating the courses is led by a global team of expert volunteer clinicians, nurses, educators, and technologists who serve as the core driving force for growth. SURGhub is anchored in the United Nations through a joint partnership agreement between the Global Surgery Foundation (GSF) and the United Nations Institute for Training and Research (UNITAR) supported by the Institute of Global Surgery in the Royal College of Surgeons in Ireland (RCSI). A project team consisting of GSF, UNITAR, and RCSI staff members provides professional project management and administration. Project funding was provided by the Johnson and Johnson Foundation.

After an extensive consultation process, three volunteer committees were established to guide platform development and operation: the Project Scope, Content, and Technical Committees. Committee members serve for a 2‐year period, with the option of renewal. In inviting membership of these committees, sex, geographic spread, and specialty representation were key considerations. Committee member specialties and locations are shown in Table 1. The Project Scope Committee is charged with delineating SURGhub's high‐level educational aims, including defining target learners and a taxonomy of content deemed to be in scope. The Content Committee defines inclusion and exclusion criteria related to content quality and appropriateness. The Technical Committee sets minimum technical and user experience standards and defines SURGhub's approach to intellectual property questions.

**TABLE 1:** SURGhub committee member specialty and location.

No.	Specialty	Location
Project scope committee		
1	Surgery (trauma)	Canada
2	Emergency medicine	Ireland
3	Surgery (general)	India
4	Surgery (general)	Rwanda
5	OB/GYN	Ireland
6	Anesthesia	Papua New Guinea
7	Anesthesia and critical care	USA
8	Nursing	Canada
Former members		
	Surgery (general)	USA
	Nursing	Zambia
Technical committee		
1	E‐learning, open‐access online material	El Salvador
2	Education, education program management	USA
3	Teaching and training specialist	Switzerland
4	Education content creation and platforms, nursing	USA
5	E‐learning, surgery (orthopedics)	UK
6	E‐learning, instructional design	Ireland
7	E‐learning, AI, technological education	Italy
8	Health technology, surgical research	USA
9	Surgery (general)	India
10	Intellectual property law	UK
Former members		
	Intellectual property law	UK
	E‐learning, e‐learning project management	UK
Content committee		
1	Surgery (general)	Kenya
2	Nurse anesthesia	USA
3	Surgery (general)	USA
4	Surgery (urology)	Nigeria
5	Surgery (pediatric)	USA
6	Surgery (general)	Mexico
7	Surgery (orthopedics)	Ethiopia
8	OB/GYN	USA
9	Anesthesia	Brazil
10	Surgery (orthopedics)	Switzerland

### 2.2 Content Curation

Initially, the SURGhub project team conducted a scoping exercise to find e‐learning courses that could potentially be appropriate for the inclusion on the platform and proactively approached numerous organizations. As the project has progressed, other organizations have increasingly approached the SURGhub team. Thus far, only asynchronous (self‐directed) e‐learning courses, including at least one assessment, have been considered for review. Courses are provided for free and with unrestricted access by academic, professional, and nongovernmental bodies. All content is hosted on the platform and remains the intellectual property of the providing institutions, which receive detailed usage feedback. Users who successfully complete each course receive a certificate of completion jointly from UNITAR and the content‐providing organization. SURGhub uses the LearnWorlds learning management system software [9] to host courses.

**FIGURE 1:**
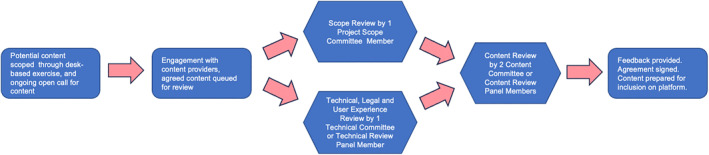
Content curation, review, and publication process.

Content submitted to SURGhub is reviewed by each of the three committees to determine whether it is appropriate for inclusion on the platform. The Technical and Content Committees are assisted in this review process by a Technical Review Panel and a Content Review Panel, respectively. All courses are reviewed by at least four experts, of whom at least two are experts in the (sub) specialty topic. Content reviewers must either practice, or have practiced, in a low‐resource environment. More than 200 expert volunteers from over 40 countries contribute as either committee members or review panel members. The content review process is shown in Figure 1.

## 3 Outcomes

**FIGURE 2:**
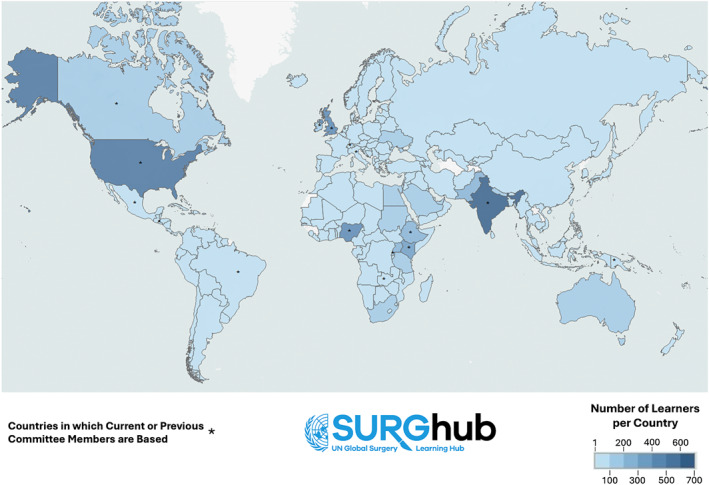
SURGhub learner and committee member map, January 20, 2025.

As of January 20, 2025, 11,451 learners were registered. Of these, 7681 provided basic demographic and professional details at the time of registration. Learners reported their location in 190 countries, as shown in Figure 2, with the majority in either low‐income countries (1205, 15.7%) or lower‐middle‐income countries (3016, 39.3%). Among the 7248 learners for whom this information was available, learners were evenly divided between those in clinical practice (53.7%, 3889) and those in education or training programs (46.3%, 3359).

Seventy‐six interactive e‐learning courses were available as shown in Table 2. All courses were available in English, with four of these also available in Spanish and two in French. Courses were provided by 20 different institutions, from both higher‐ and lower‐resource settings across the world. Course length varied significantly, from single topic courses, which could be completed in an hour, to comprehensive courses intended to accompany learners through training over multiple years. Courses were categorized as follows, with some courses in multiple categories: anesthesia (41 courses), surgery (35), perioperative nursing (15), obstetrics/gynecology (11), and nontechnical skills (10).

**TABLE 2:** Courses on SURGhub, January 20, 2025.

No.	Course	Provided by
English language		
1	Difficult Airway Algorithm 2023	American Society of Anesthesiologists
2	Drug Errors in Anesthesia 2023	American Society of Anesthesiologists
3	Emergency checklists in anesthesia practice	American Society of Anesthesiologists
4	Infection Control and Prevention 2023	American Society of Anesthesiologists
5	Z‐plasty	AmoSmile
6	Central venous access devices (CVADs)	Ausmed
7	CVAD complications: Prevention and management:	Ausmed
8	Postoperative assessment and monitoring	Ausmed
9	2‐handed knot‐tying (right and left handed)	Behind the Knife
10	1‐handed knot‐tying (right and left handed)	Behind the Knife
11	Surgical skills—suturing—horizontal mattress	Behind the Knife
12	Surgical skills—suturing—vertical mattress	Behind the Knife
13	Research methodology course	College of Anesthesiologists of East, Central and Southern Africa
14	Surgical foundations	College of Surgeons of East, Central and Southern Africa
15	Hemorrhage control in prehospital settings	CrashSavers Team
16	Breast cancer surgery	ecancer
17	Colon cancer surgery	ecancer
18	Pancreatic cancer surgery	ecancer
19	Rectal cancer	ecancer
20	Perioperative Nursing E‐Learning Foundational Programme	East, Central and Southern Africa College of nursing and Midwifery
21	Navigating the global surgery ecosystem	Global Surgery Foundation
22	Wound healing after C‐section	Health Professional Academy
23	Essential burn care (EBC)	Interburns
24	Pulse oximetry in the context of COVID‐19	Lifebox
25	Pulse oximetry	Lifebox
26	Personal protective equipment	Lifebox
27	Trauma and Disaster Team Response Course	McGill University center for global surgery
28	Arterial blood pressure measurement	PerioperativeCPD
29	Understanding capnography	PerioperativeCPD
30	Understanding LAST	PerioperativeCPD
31	Safe clinical handover	Royal College of Surgeons in Ireland
32	Video consultations	Royal College of Surgeons in Ireland
33	Communication skills for telephone consultations	Royal College of Surgeons in Ireland
34	Patient‐centerd care	Royal College of Surgeons in Ireland
35	Common intraoperative problems	ReSurge International
36	Hand trauma reconstruction	ReSurge International
37	A crash course in facial fractures	ReSurge International
38	Microsurgery workhorse flaps	ReSurge International
39	Burns: wound care and pain management	ReSurge International
40	Pressure sore reconstruction	ReSurge International
41	Vascular malformations	ReSurge International
42	Scalp reconstruction	ReSurge International
43	Lip and cheek reconstruction	ReSurge International
44	Ear reconstruction	ReSurge International
45	Overview of skin cancer	ReSurge International
46	Cleft lip and palate	ReSurge International
47	Nasal reconstruction	ReSurge International
48	Breast reconstruction	ReSurge International
49	Facial paralysis	ReSurge International
50	Acute burn care	ReSurge International
51	Oral health in comprehensive cleft care: oral health professionals	Smile Train
52	Essential emergency and critical care	Stanford University Learning Resource Center
53	Fascia iliaca compartment block: landmark and ultrasound approach	World Federation of Societies of Anesthesiologists
54	Anesthesia for caesarean delivery	World Federation of Societies of Anesthesiologists
55	Transversus abdominis plane block	World Federation of Societies of Anesthesiologists
56	Blood sparing techniques in pediatric anesthesia	World Federation of Societies of Anesthesiologists
57	Lumbar plexus block—landmark technique (PSOAS compartment block)	World Federation of Societies of Anesthesiologists
58	Anesthetic considerations for vaginal birth after caesarean delivery	World Federation of Societies of Anesthesiologists
59	The labor epidural: troubleshooting	World Federation of Societies of Anesthesiologists
60	Rapid sequence induction	World Federation of Societies of Anesthesiologists
61	Key concepts in the perioperative management of spinal cord injuries	World Federation of Societies of Anesthesiologists
62	Ultrasound guided femoral nerve block	World Federation of Societies of Anesthesiologists
63	Perioperative blood management in the pediatric patient	World Federation of Societies of Anesthesiologists
64	Conversion of labor epidural analgesia to anesthesia for cesarean Delivery	World Federation of Societies of Anesthesiologists
65	Tranexamic acid	World Federation of Societies of Anesthesiologists
66	Fascia iliaca compartment block: an update	World Federation of Societies of Anesthesiologists
67	Anesthesia for tissue free flap surgery	World Federation of Societies of Anesthesiologists
68	Practical considerations to access the epidural space	World Federation of Societies of Anesthesiologists
69	Principles of regional anesthesia in children	World Federation of Societies of Anesthesiologists
70	Anesthesia for posterior cranial fossa surgery	World Federation of Societies of Anesthesiologists
71	Obstetrics in the setting of remote retrieval medicine	World Federation of Societies of Anesthesiologists
72	Nerve stimulation for peripheral nerve blockade	World Federation of Societies of Anesthesiologists
73	Anesthesia for craniotomy and brain tumor resection	World Federation of Societies of Anesthesiologists
74	Adjuvant medications for peripheral nerve block	World Federation of Societies of Anesthesiologists
75	Speaking up in the operating room: escalating concerns	World Federation of Societies of Anesthesiologists
76	Perioperative medicine: an overview	World Federation of Societies of Anesthesiologists
Spanish language		
77	Conversión de Analgesia Epidural para Labor a Anestesia para Operación Cesárea	World Federation of Societies of Anesthesiologists
78	Anestesia para Craneotomía y Resección de Tumor Cerebral	World Federation of Societies of Anesthesiologists
79	Consideraciones Anestésicas para el Parto Vaginal luego de Cesárea	World Federation of Societies of Anesthesiologists
80	Epidural en el Trabajo de Parto: Solución de Problemas	World Federation of Societies of Anesthesiologists
French language		
81	Intervention de Traumatologie et Catatrophe en Equipe	McGill University Center for Global Surgery
82	Acide Tranexamique	World Federation of Societies of Anesthesiologists

Learners provided qualitative and quantitative feedback at the end of each course (*n* = 4621); the median overall course rating was 4.6/5. Qualitative feedback was markedly positive, with high content relevance to real‐world practice a consistent theme. The most common suggestion for improvement was the inclusion of further learning resources on course topics, such as textbooks and academic resources. Courses with higher ratings were often praised for their use of video and interactive elements, whereas feedback for lower‐rated courses frequently included suggestions to incorporate more multimedia and engagement tools. Technical challenges were explicitly solicited, but few learners reported challenges. Total platform usage time was 82,150 h, with 4190 course completion certificates issued. It is notable that courses authored in low‐resource settings had high levels of usage.

## 4 Discussion

SURGhub represents a transformative advancement in global surgical education, distinguished by its innovative participatory approach to addressing critical gaps in accessibility and knowledge dissemination. Unlike existing online learning platforms, SURGhub specifically targets the unique challenges faced by underserved surgical learners worldwide through three key differentiators: free access to comprehensive educational resources, low‐bandwidth optimization for regions with limited internet connectivity, and culturally informed content delivery that reflects diverse geographical contexts.

The platform's early adoption metrics and user feedback demonstrate strong potential for scale up and impact, while simultaneously highlighting strategic imperatives for continued growth. To fully realize its potential, SURGhub must expand its educational offerings across multiple dimensions. This includes incorporating new types of content, such as surgical atlases, implementing advanced search functionality, creating personalized learning pathways, and establishing interactive learning communities. Furthermore, the platform must overcome language barriers by providing multilingual content to ensure truly global accessibility.

Critical to SURGhub's evolution is a robust impact assessment framework. This framework must address several key questions: What is the demographic composition of our active user base and which new audiences should be reached? How effectively does our content align with local clinical needs? What role does English language proficiency play in platform engagement? Most importantly, how does SURGhub's educational content translate into measurable improvements in local clinical practice and patient outcomes? The answers to these questions will drive continuous platform refinement and inform strategic decisions. Successful open‐access medical education platforms, such as OPENPediatrics [10] and Open Critical Care [11], have demonstrated the viability of this approach, providing valuable precedents for SURGhub's development.

As a platform that curates preexisting content, SURGhub is necessarily limited by the availability of such content—available content is unlikely to cover every aspect of every targeted specialty and subspecialty and some duplication is inevitable. SURGhub also faces substantial operational and financial challenges, particularly in maintaining a no‐cost model for both content providers and learners without relying on advertising revenue. However, these challenges present compelling investment opportunities for stakeholders committed to advancing equitable surgical education globally. The platform's scalable infrastructure and demonstrated impact position it favorably for sustainable growth. With continued support from the global surgical community and strategic partners, SURGhub is well‐positioned to address its current challenges and capitalize on emerging opportunities.

## 5 Conclusion

SURGhub is establishing new benchmarks in open‐access education for surgical providers by directly addressing the needs of underserved learners through innovative, participatory technological solutions. As the platform continues to evolve and expand its reach, it demonstrates the potential to fundamentally transform global surgical education accessibility and quality. This positions SURGhub as a pivotal force in advancing health equity through education.

## Author Contributions

**Eric O’Flynn:** conceptualization, writing – original draft, writing – review and editing, visualization, formal analysis, data curation, project administration. **Musliu Adetola Tolani:** conceptualization, writing – original draft, writing – review and editing. **Emilie Joos:** conceptualization, writing – original draft, writing – review and editing. **Jean O’Sullivan:** writing – review and editing, writing – original draft, conceptualization. **Dhananjaya Sharma:** conceptualization, writing – original draft, writing – review and editing. **Sherry M. Wren:** conceptualization, writing – original draft, writing – review and editing. **Ainhoa Costas Chavarri:** conceptualization, writing – original draft, writing – review and editing. **Pauline B. Wake:** conceptualization, writing – original draft, writing – review and editing. **Andrea S. Parker:** conceptualization, writing – original draft, writing – review and editing. **Jackie Rowles:** conceptualization, writing – original draft, writing – review and editing. **Lubna Khan**: conceptualization, writing – original draft, writing – review and editing. **Sara M. Mohan:** conceptualization, writing – original draft, writing – review and editing. **Sabina M. Siddiqui:** conceptualization, writing – original draft, writing – review and editing. **Adolfo Leyva‐Alvizo:** conceptualization, writing – original draft, writing – review and editing. **Richard O. E. Gardner:** conceptualization, writing – original draft, writing – review and editing. **Rahel Nardos:** conceptualization, writing – original draft, writing – review and editing. **Luiz Fernando dos Reis Falcão:** conceptualization, writing – original draft, writing – review and editing. **Claude Martin Jr**: conceptualization, writing – original draft, writing – review and editing. **Somprakas Basu:** conceptualization, writing – original draft, writing – review and editing. **Emilio Velis:** conceptualization, writing – original draft, writing – review and editing. **Anna M. Darelli-Anderson:** conceptualization, writing – original draft, writing – review and editing. **Vincenzo Palatella:** conceptualization, writing – original draft, writing – review and editing. **Andrew Katz:** conceptualization, writing – original draft, writing – review and editing. **Rebecca Silvers:** conceptualization, writing – original draft, writing – review and editing. **Harry Papadopoulos:** conceptualization, writing – original draft, writing – review and editing. **Advait J. Gandhe:** conceptualization, writing – original draft, writing – review and editing. **Elizabeth Khvatova:** conceptualization, writing – original draft, writing – review and editing. **Karina Olivo:** conceptualization, writing – original draft, writing – review and editing. **Michelle N. Odonkor:** conceptualization, writing – original draft, writing – review and editing. **Arushi Biswas:** conceptualization, writing – original draft, writing – review and editing. **Francesca Vitucci:** conceptualization, writing – original draft, writing – review and editing, visualization, formal analysis, data curation. **Léa Simon:** conceptualization, writing – original draft, writing – review and editing, visualization, formal analysis, data curation. **Ines Perić:** conceptualization, writing – original draft, writing – review and editing, visualization, data curation, formal analysis. **Sebastian Hofbauer:** conceptualization, writing – original draft, writing – review and editing. **Noor Alesawy:** conceptualization, writing – original draft, writing – review and editing. **Juan Carlos Puyana:** conceptualization, writing – original draft, writing – review and editing. **Geoffrey Ibbotson:** conceptualization, writing – original draft, writing – review and editing, supervision.

## Ethics Statement

Data provided in this article are either available in the public domain or is nonsensitive educational research, which is unlikely to adversely impact learners, and thus does not require research ethics review.

## Conflicts of Interest

Sherry M. Wren is an editor‐in‐chief of the World Journal of Surgery. All other authors declare that they have no conflicts of interest.

## Data Availability

The data that support the findings of this study are available from the corresponding author upon reasonable request.

## References

[1] M. G. Shrime , S. W. Bickler , B. C. Alkire , and C. Mock , “Global Burden of Surgical Disease: An Estimation From the Provider Perspective,” Lancet Global Health 3 (2015): S8–S9, 10.1016/S2214-109X(14)70384-5. 25926322

[2] J. G. Meara , A. J. M. Leather , L. Hagander , et al., “Global Surgery 2030: Evidence and Solutions for Achieving Health, Welfare, and Economic Development,” Lancet 386, no. 9993 (2015): 569–624, 10.1016/S0140-6736(15)60160-X. 25924834

[3] E. O’Flynn , A. Ahmed , A. Biswas , N. Bempong‐Ahun , I. Perić , and J. C. Puyana , “E‐Learning Supporting Surgical Training in Low‐Resource Settings,” Current Surgery Reports 12, no. 6 (2024): 151–159, 10.1007/s40137-024-00399-8.

[4] S. Barteit , D. Guzek , A. Jahn , T. Bärnighausen , M. M. Jorge , and F. Neuhann , “Evaluation of E‐Learning for Medical Education in Low‐ and Middle‐Income Countries: A Systematic Review,” Computers & Education 145 (2020): 103726, 10.1016/j.compedu.2019.103726. 32565611 PMC7291921

[5] C. van Zyl , M. Badenhorst , S. Hanekom , and M. Heine , “Unravelling ‘Low‐Resource Settings’: A Systematic Scoping Review With Qualitative Content Analysis,” BMJ Global Health 6 (2021): e005190, 10.1136/bmjgh-2021-005190. PMC8183220 34083239

[6] The World Bank . World Bank Country and Lending Groups, (2024), https://datahelpdesk.worldbank.org/knowledgebase/articles/906519‐world‐bank‐country‐and‐lending‐groups.

[7] H. Holmer , A. Lantz , T. Kunjumen , et al., “Global Distribution of Surgeons, Anaesthesiologists, and Obstetricians,” Lancet Global Health 3 (2015): S9–S11, 10.1016/S2214-109X(14)70349-3. 25926323

[8] S. Sheik Ali , Z. Jaffry , M. N. Cherian , et al., “Surgical Human Resources According to Types of Health Care Facility: An Assessment in Low‐ and Middle‐Income Countries,” World Journal of Surgery 41, no. 11 (2017): 2667–2673, 10.1007/s00268-017-4078-4. 28608018 PMC5643366

[9] LearnWorlds . LearnWorlds Learning Management System Software (2024), https://www.learnworlds.com.

[10] OPENPediatrics . OPENPediatrics (2024), https://www.openpediatrics.org.

[11] OpenCriticalCare . The OpenCriticalCare.Org Project (2024), https://opencriticalcare.org.

